# Feasibility of Near-Infrared Spectroscopy in the Classification of Pig Lung Lesions

**DOI:** 10.3390/vetsci11040181

**Published:** 2024-04-18

**Authors:** Maria Olga Varrà, Mauro Conter, Matteo Recchia, Giovanni Loris Alborali, Antonio Marco Maisano, Sergio Ghidini, Emanuela Zanardi

**Affiliations:** 1 Department of Food and Drug, University of Parma, Strada del Taglio 10, 43126 Parma, Italy; mariaolga.varra@unipr.it (M.O.V.); emanuela.zanardi@unipr.it (E.Z.); 2 Department of Veterinary Science, University of Parma, Strada del Taglio 10, 43126 Parma, Italy; 3 Istituto Zooprofilattico Sperimentale della Lombardia e dell’Emilia-Romagna-Headquarters, Via A. Bianchi, 9, 25124 Brescia, Italy; giovanni.alborali@izsler.it (G.L.A.); antoniomarco.maisano@izsler.it (A.M.M.); 4 Department of Veterinary Medicine and Animal Sciences, Via dell’Università 6, 26900 Lodi, Italy; sergio.ghidini@unimi.it

**Keywords:** NIR spectroscopy, pneumonia, porcine respiratory disease, necropsy, veterinary diagnostic

## Abstract

**Simple Summary:**

Accurate classification of lung lesions detected in porcine respiratory disease outbreaks is a prerequisite in the overall diagnostic process and greatly contributes to appropriate disease management. The use of rapid and non-destructive techniques during necropsy, such as near-infrared spectroscopy, could enhance the achievement of this objective. In this view, the aim of the present study was to investigate the potential of such a technique in differentiating between normal, congested, and pathological pig lung tissue and between different patterns of pulmonary inflammation. Overall, an effective discrimination between normal and pathological lung tissue was observed, with congested tissues exhibiting an intermediate behavior. Remarkable classification performances were also attained for the identification of the different pathological lesions, demonstrating high levels of accuracy, precision, and sensitivity. In conclusion, these findings underscore the promising utility of near-infrared spectroscopy as an invaluable tool in veterinary diagnostics.

**Abstract:**

Respiratory diseases significantly affect intensive pig farming, causing production losses and increased antimicrobial use. Accurate classification of lung lesions is crucial for effective diagnostics and disease management. The integration of non-destructive and rapid techniques would be beneficial to enhance overall efficiency in addressing these challenges. This study investigates the potential of near-infrared (NIR) spectroscopy in classifying pig lung tissues. The NIR spectra (908–1676 nm) of 101 lungs from weaned pigs were analyzed using a portable instrument and subjected to multivariate analysis. Two distinct discriminant models were developed to differentiate normal (N), congested (C), and pathological (P) lung tissues, as well as catarrhal bronchopneumonia (CBP), fibrinous pleuropneumonia (FPP), and interstitial pneumonia (IP) patterns. Overall, the model tailored for discriminating among pathological lesions demonstrated superior classification performances. Major challenges arose in categorizing C lungs, which exhibited a misclassification rate of 30% with N and P tissues, and FPP samples, with 30% incorrectly recognized as CBP samples. Conversely, IP and CBP lungs were all identified with accuracy, precision, and sensitivity higher than 90%. In conclusion, this study provides a promising proof of concept for using NIR spectroscopy to recognize and categorize pig lungs with different pathological lesions, offering prospects for efficient diagnostic strategies.

## 1. Introduction

Porcine respiratory disease complex (PRDC) is a major cause of production losses and antimicrobial consumption in pig farming, with serious implications for animal health and welfare. The combination of environmental and management stressors and genetic factors impairs the pulmonary and systemic defense mechanisms of pigs, leading to pneumonia with polymicrobial etiology [1]. Viruses such as porcine reproductive and respiratory syndrome virus (PRRSV), swine influenza virus (SIV), porcine circovirus type 2 (PCV2), pseudorabies virus (PRV), along with *Mycoplasma hyopneumoniae* and *Actinobacillus pleuropneumoniae* typically act as primary pathogens, promoting secondary bacterial infections by *Pasteurella multocida*, *Trueperella pyogenes*, *Mycoplasma* spp., *Glaesserella parasuis*, and *Streptococcus suis* [2,3]. Lung pathology is thus determined by the infectious agents involved, as well as by the efficacy of host defenses and pharmacological treatments.

Due to its multifactorial nature, the management of PRDC requires a multidisciplinary approach. Clinical information, epidemiological data, and direct or indirect in vivo diagnostic results should be integrated with post-mortem examinations [4,5]. Gross morpho-pathological exams are helpful in selecting the most appropriate laboratory tests, such as bacteriological, virological, and molecular analyses [6]. Indeed, the categorization of lung lesions based on gross morphological patterns may help to narrow the list of differential diagnoses, although mixed pathological scenarios often occur, and single pathogens may be associated with different types of lesions [4]. Furthermore, histopathology can provide more accurate information regarding the pathological process observed and, in some cases, confirm the causative association between lesions and specific pathogens [4].

Among morpho-pathological patterns of lung inflammation, catarrhal bronchopneumonia (CBP), fibrinous pleuropneumonia (FPP), and interstitial pneumonia (IP) are frequently found at necropsy. CBP in pigs is typical of *M. hyopneumoniae* pulmonary infection, although other pathogens may cause similar lesions [7]. FPP is most commonly associated with *A. pleuropneumoniae* lung infection, as well as with *Actinobacillus suis* or virulent strains of *P. multocida* responsible for pleuritis [4,8]. Finally, IP is typical of viral infections, with PRRSV and PCV2 commonly implicated [6].

The ability to differentiate pathological patterns quickly and accurately is crucial for selecting the proper diagnostic tests. While macroscopic and histological examinations contribute to the formulation of a morphological diagnosis, the availability of objective, non-destructive, automatable, and rapid analytical techniques to obtain confirmation in a short time could be extremely useful. The implementation of such techniques within veterinary diagnostic labs and slaughterhouses would expedite and optimize meat inspection and diagnostic processes, given the possibility of complementing or partially replacing extensive human labor, and would enhance the overall efficiency of animal disease management. Moreover, these tools could play a pivotal role in enhancing animal health and welfare monitoring within pig abattoirs. Indeed, the slaughterhouse represents a strategic control point to assess the impact of respiratory disease in pig fattening farms [9,10,11]. Over the years, several scoring methods have been proposed to quantify pulmonary lesions in slaughtered pigs, providing farmers and veterinarians with reports on the effectiveness of on-farm prevention and management strategies [12,13,14]. These scoring systems evaluate the extent and severity of lung lesions by visual inspection and palpation, particularly in relation to bronchopneumonia and chronic pleuritis [15]. In such contexts, the adoption of innovative non-destructive methods and technologies in slaughterhouses, as promoted by Regulation (EU) 625/2017 [16], would not only enhance official veterinary controls to safeguard food safety and public health but also bolster the role of abattoirs as animal welfare and epidemiological observatories [17,18]. Moreover, techniques capable of classifying the type of pulmonary inflammation in a continuous and standardized manner would provide an overview of the respiratory health status of a farm and more precise feedback to herd veterinarians, thus enhancing the on-farm management of PRDC [19].

Near-infrared (NIR) spectroscopy has emerged as a promising tool with a proven track record in diverse fields such as agriculture, food, and pharmaceuticals. This technique is based on the fundamental principle of measuring the interaction between NIR electromagnetic radiation and the molecules within a sample matrix. This interaction results in a unique spectral fingerprint that includes comprehensive information related to the composition and condition of the analyzed tissues [20]. Theoretically, this spectral fingerprint could be used to rapidly recognize distinctive patterns specific to lung lesions, facilitating efficient and accurate identification of pathological features within a short time frame and improving the overall costs of operation.

Of note, NIR spectroscopy has gained increasing recognition in human medical research [21], particularly for screening pathological kidney tissues and conditions associated with fibrotic and steatotic liver diseases [22,23,24]. The versatility of this technique has also been proven in veterinary medicine and meat inspection, where NIR and ultraviolet-visible (UV) spectroscopy, often integrated within hyperspectral imaging systems, have been successfully employed to differentiate normal from septicemic poultry carcasses, discriminate among different poultry myopathies [25,26,27,28], identify milk spot livers [29], and discern between healthy and pathological sheep lung tissues [30].

Based on this premise, the primary goal of this study was to explore for the first time the use of NIR spectroscopy, specifically employing a portable device, as an innovative solution for the assessment of lung lesions in pigs, thereby providing a groundbreaking proof of concept regarding its potential usefulness within this specific field.

The outcomes detailed in the present manuscript represent the first promising achievement of an ongoing research project dedicated to the development and application of objective measurement systems based on near-infrared spectroscopy and computer vision. These systems aim to support postmortem inspection of pig carcasses and organs, as well as diagnostic procedures.

## 2. Materials and Methods

### 2.1. Lung Samples 

A total of 101 lungs, sampled from weaned pigs (weighing less than 50 kg) delivered to the Diagnostic Section of the “Istituto Zooprofilattico Sperimentale della Lombardia e dell’Emilia Romagna” (IZSLER) in Brescia (Italy) for postmortem investigations, were included in the present study. Only well-preserved lungs were evaluated to avoid the inclusion of tissues with potential postmortem alterations.

Expert veterinarians conducted a comprehensive examination of each lung, involving visual inspection and palpation, aimed to establish a gross morphological diagnosis. Three acute pneumonia patterns, namely catarrhal bronchopneumonia (CBP), fibrinous pleuropneumonia (FPP), and interstitial pneumonia (IP), were considered based on the classification scheme proposed by Ruggeri et al. [6]. Briefly, CBP is characterized by a cranioventral distribution, typically affecting the apical, middle, and cranial portions of the diaphragmatic lung lobes. In acute cases, affected parenchyma is consolidated and dark red-purple in color. On the cut surface, scarce to abundant whitish catarrhal exudate can be observed filling the airways. In acute FPP, lesions are mainly located in the dorso-caudal portions of the diaphragmatic lobes. Affected areas appear dark reddish-purple to black and slightly to moderately firm. On the cut surface, the parenchyma appears extremely heterogeneous with diffuse hemorrhage, distended interlobular septa, and areas of friable necrosis, usually surrounded by white lines composed of leukocytes and fibrin. Layers of fibrin can be observed on the pleural surface. Finally, IP is commonly characterized by diffuse lesion distribution. Lungs typically fail to collapse, have a rubbery consistency, and their color changes from light to purple-red. Acute alterations also include diffuse hyperemia and severe interlobular edema.

**Figure 1:**
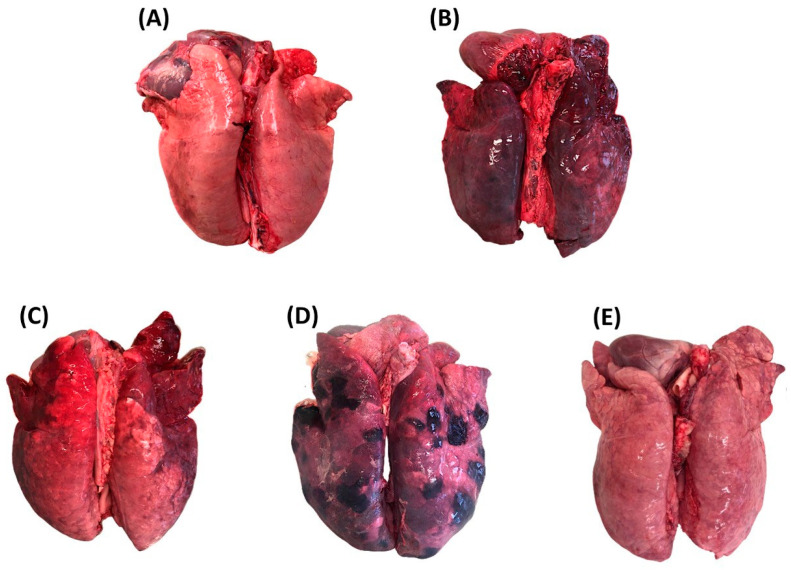
Lung tissue categories selected at necropsy. (**A**) normal lung tissue; (**B**) congested lung tissue; (**C**) catarrhal bronchopneumonia; (**D**) fibrinous pleuropneumonia; (**E**) interstitial pneumonia).

When available, normal (i.e., healthy, N) and congested (C) portions of tissue found within the same pathological lung or from distinct (non-pathological) lungs were also included. Lung tissue was classified as congested if its consistency was unchanged and its color was darker red compared to normal parenchyma. Representative images of the selected lung tissue categories are reported in Figure 1.

### 2.2. Near-Infrared Spectroscopic Analysis 

NIR diffuse reflectance spectra of lung tissue samples were recorded by expert veterinarians during necropsy at room temperature (20 °C) using a battery-powered portable device (MicroNIR OnSite-W, Viavi Solutions, Santa Rosa, CA, USA) in the 908–1676 nm infrared region (6.1 nm apparent resolution, 10 ms integration time, 100 co-added scans). The calibration of the instrument and the background signal correction were performed before sample analysis by the periodical recording (every ten lung tissue samples) of total absorbance (i.e., “black”) and total reflectance (i.e., “white”) reference spectra. To this purpose, the Spectralon^®^ Diffuse Reflectance Standards (Labsphere, Inc., North Sutton, NH, USA) was employed.

**Figure 2:**
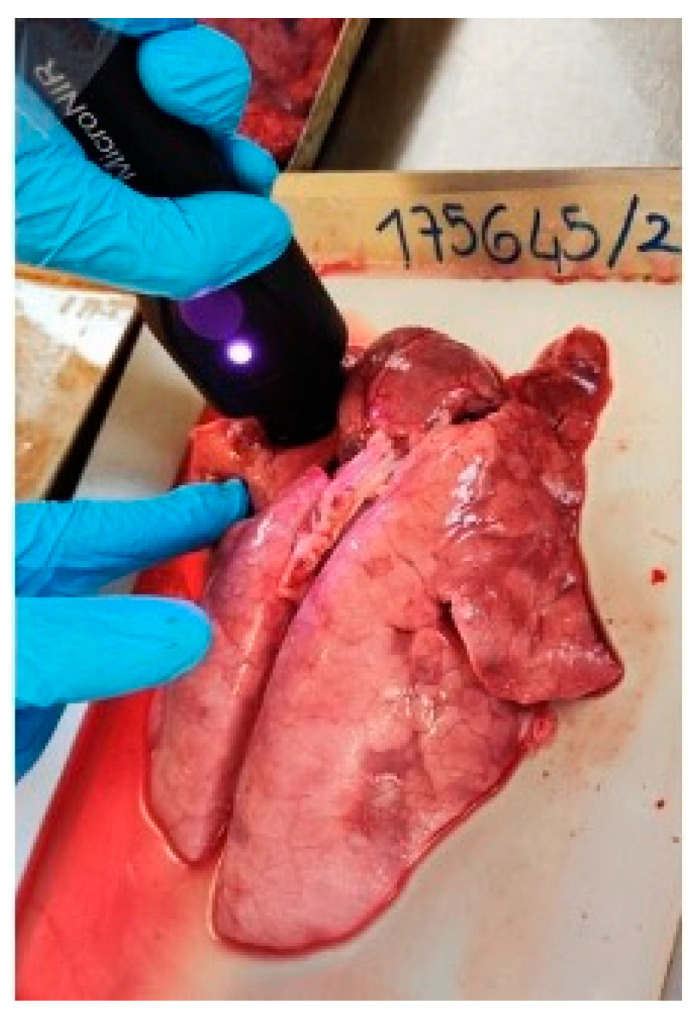
NIR spectra recording of pig lung tissues using the portable spectrometer.

All the visible and well-defined N, C, or pathological (P) areas of each lung were scanned by aligning the instrument at a right angle to the surface of the organ (scanning area of approximately 1.5–2 cm^2^), as represented in Figure 2. For each analyzed sample area, eight replicate spectra were recorded to capture a sufficient amount of variability associated with the inherent compositional inhomogeneity of the lung tissue. This was achieved by rotating the instrument between consecutive spectrum acquisitions.

### 2.3. NIR Data Processing 

Before conducting data analysis and the development of discriminant models, a thorough inspection and visual assessment of all NIR spectra were carried out to find and exclude potential analytical outliers and divergent samples. The filtered NIR data matrix subjected to elaboration by multivariate statistics included a total of N = 1298 spectra (distributed into different lung tissue categories according to Table 1) and 125 variables (corresponding to absorbance values of each sample at the considered NIR wavelengths). 

The selected raw NIR spectra were then mean centered and pre-processed to enhance the quality, accuracy, and robustness of the subsequent data processing steps. Indeed, the raw NIR spectra, while holding valuable information, exhibited several undesirable characteristics (i.e., baseline shifts and drifts), which hampered the identification of small spectral features crucial for discriminating lung tissues. Two pre-processing techniques, commonly used in the NIR spectroscopy analytical workflow, were concatenated and applied to the raw spectra:

- Standard normal variate (SNV): useful to mitigate the impact of light scattering and reduce baseline shifts/drifts;
- Fourth-order derivative (4Der): useful to increase the resolution among overlapping peaks and highlight spectral differences.

### 2.4. Multivariate Statistics 

Multivariate analysis, consisting of Orthogonal Partial Least Squares Discriminant Analysis (OPLS-DA), was then applied to the SNV + 4Der pre-processed spectral data in order to develop classification models that could discriminate between the different categories of lung tissues. More specifically, OPLS-DA was used to establish a correlation between the NIR spectral data and the results from the gross anatomopathological diagnosis performed by veterinarians (serving as the reference standard for assigning classes to the lung samples).

Specifically, two separate OPLS-DA models were developed:

- A 3-class model (i.e., “Model 1”), created with the objective of distinguishing between N, C, and P lung tissues;
- A 3-class model (i.e., “Model 2”), including only spectra from pathological tissues and designed to discriminate among CBP, FPP, and IP lung lesions.

Before the construction of the models, the whole spectral dataset was prepared by randomly splitting it into calibration (75%) and validation subsets (25%). This procedure was performed both for the OPLS-DA Model 1 and Model 2. By consequence, the calibration sets, containing the majority of the spectra, included N = 974 spectra for Model 1 and N = 441 spectra for Model 2. The calibration sets were employed for model training and development, which were performed by using a 7-fold cross-validation.

The validation sets, including the remaining spectra not used for calibration (i.e., N = 324 spectra for Model 1 and N = 147 spectra for Model 2), were instead employed for independent testing (external validation) of the developed calibration OPLS-DA models. They served as unbiased sample sets to evaluate the overall performances of the models in discriminating N vs. C vs. P or FPP vs. CBP vs. IP lung tissues when applied to new unknown lung samples.

Following the external validation stage, confusion matrices were generated for both Model 1 and Model 2 to summarize the predicted vs. the actual classifications of the samples included in the two validation spectral subsets. Starting from the confusion matrices, true-positive, true-negative, false-positive, and false-negative samples for each class of lung samples were identified. These values were then used to calculate specificity, sensitivity, accuracy, and precision percentage values, providing comprehensive performance metrics for the discriminant models [31].

Finally, to identify the crucial NIR wavelengths that had a significant impact on predicting the class membership of the lung samples, the Variable Importance in Projection (VIP) index was employed, where VIP values equal to or greater than 1 were considered significant [32].

Multivariate analysis was performed by using SIMCA^®^ software v. 17.0.2.34594 (Sartorius Stedim Data Analytics AB, Umea, Sweden).

## 3. Results

### 3.1. NIR Spectral Profiles of Lung Tissues 

**Figure 3:**
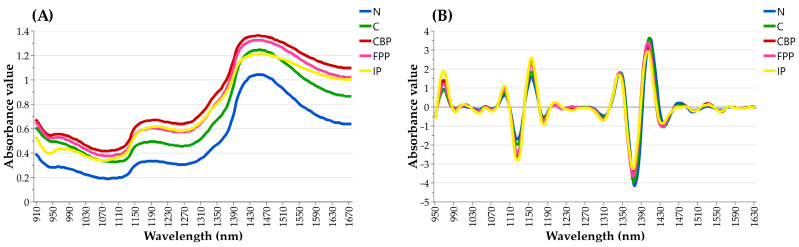
Comparative representation of average NIR spectra of the analyzed lung tissue classes, with (**A**) displaying raw NIR spectra and (**B**) illustrating corrected NIR spectra after the application of SNV + 4Der pre-processing techniques.

To enhance visualization and emphasize spectral characteristics, the average NIR spectra for each analyzed class of lung tissues (i.e., N, C, P, CBP, FPP, and IP) were calculated and examined. The raw and pre-processed (SNV + 4Der) average NIR spectra, spanning the 908–1676 nm NIR region, are depicted in Figure 3A and Figure 3B, respectively. 

Upon examination, unwanted baseline drifts, shift effects, and the presence of prominent unresolved peaks were observed in the raw spectra, rendering them uninformative and useless for direct analysis (Figure 3A). In contrast, the pre-processed spectra were found to be characterized by improved resolution and suitable for discriminant analysis (Figure 3B). The graphical evaluation of SNV + 4Der spectra allowed for the identification of spectral regions where peaks tended to exhibit variations in absorbance values among different lung tissue categories. As observed in Figure 3B, the IP lung tissues exhibited higher absorption values in the peaks located within the 950–990 nm, 1070–1110 nm, and 1140–1190 nm NIR regions. In contrast, absorption bands within the 1320–1360 nm and 1400–1430 nm NIR regions were found to be higher in N and C lung tissues compared to pathological ones (Figure 3B). Additionally, minor absorption peaks were detected around 1460–1490 nm and 1520–1550 nm, although these showed similar intensities across all investigated lung categories.

### 3.2. Discriminant Analysis

Model 1, aimed at distinguishing among N, C, and P lung tissues, was constructed by applying OPLS-DA to the calibration subset. This model was found to be characterized by a total of 2 predictive and 7 orthogonal components. As summarized in Table 2, the cumulative goodness-of-fit (R^2^X_cum_) and the cumulative goodness-of-prediction (R^2^Y_cum_ and Q^2^_cum_) parameters of this model were globally higher than 0.5, thus suggesting its suitability for the intended purpose of discriminating N vs. C vs. P lung tissues.

**Figure 4:**
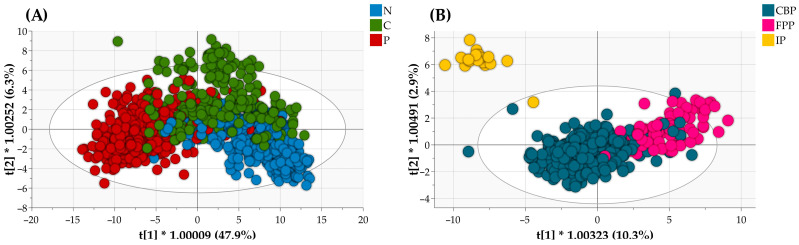
OPLS-DA score scatter plots of Model 1 for the discrimination of N, C, and P lung tissues (**A**) and of Model 2 for the discrimination of CBP, FPP, and IP lung tissues (**B**) resulting from (internal) cross-validation of the NIR spectra belonging to the calibration sets.

The score scatter plot, serving as visual representations of individual tissue samples in a bi-dimensional plane defined by the first two predictive components extracted from Model 1, displayed quite uniform clusters for each lung tissue category (Figure 4A). However, while the three clusters were generally well defined, a significant degree of overlap was observed among samples from different categories.

Model 2, designed for the discrimination among CBP, FPP, and IP, was developed from the calibration subset using a total of 2 predictive and 7 orthogonal components. The model displayed an R^2^X_cum_ of 0.904, an R^2^Y_cum_ of 0.647, and a Q^2^_cum_ of 0.615 (Table 2). Compared to Model 1, the fitting ability was hence found to be lower, but the prediction ability was enhanced.

As illustrated in the OPLS-DA score scatter plot of Model 2 presented in Figure 4B (bounded by the first two predictive components derived from the model, t[1] and t[2]), distinct sample clusters corresponding to the three different patterns of pneumonia emerged. Of note, the separation of IP samples was particularly pronounced, despite their limited numerical representation compared to other groups. IP samples were indeed positioned distinctly apart from CBP and FPP samples along both the t[1] and t[2], with only one sample closely overlapping with the samples of the CBP group (Figure 4B). Even in this case, it is crucial to emphasize that a certain degree of overlap was evident among CBP and FPP samples (Figure 4B).

Table 3 presents the predictive VIP values extracted from the two discriminant models for distinguishing between different lung tissue conditions and pneumonia types using NIR spectroscopy. For Model 1, VIP values at specific NIR wavelengths highlighted the importance of amide/protein (1515 nm, VIP = 1.28), water (976 nm, VIP = 1.27), and alkyl alcohol (964 nm, VIP = 1.23) in discriminating between N, C, and P lung tissues. The influence of amines and fatty acids was also evident, due to high VIP values at 1472, 1521, 1391, 1428, 1422, and 1327 nm NIR wavelengths (Table 3). In Model 2, the highest VIP values were observed at 1580 nm (alcohol/water, VIP = 2.24), 1453 nm (water, VIP = 2.16), and 1471 nm (amide/protein, VIP = 2.06), emphasizing the significance of patterns associated with molecules in differentiating between CBP, FPP, and IP lungs. Also in this case, the molecular patterns associated with aliphatic hydrocarbons typical of fatty acids were evident (Table 3).

Following the external validation of Model 1 using samples of the validation subset, promising results were achieved, indicating the model’s potential to achieve relatively high correct classification rates (i.e., precision) toward unknown samples belonging to different tissue categories: 84.6% N lungs, 69.9% for C lungs, and 90.5% for P lungs, as observed in the confusion matrix reported in Table 4. However, a notable challenge emerged during external validation, consistent with the observations from the score plot reported in Figure 4A. Specifically, C lungs exhibited a higher misclassification rate, frequently being inaccurately categorized as either N or P (Table 4).

**Figure 5:**
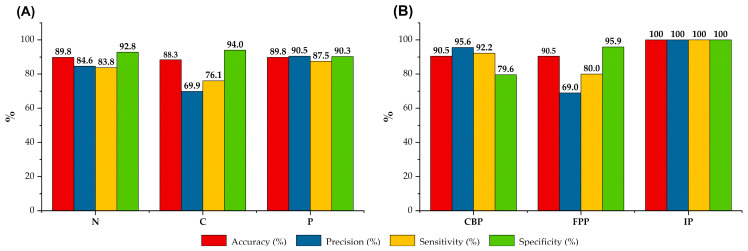
Accuracy, precision, sensitivity, and specificity percentage values resulting from external validation for each class of lung tissues of discriminant Model 1—normal (N) vs. congested (C) vs. pathological (P) lungs (**A**) and discriminant Model 2—lungs affected by catarrhal bronchopneumonia (CBP) vs. fibrinous pleuropneumonia (FPP) vs. interstitial pneumonia (IP) (**B**).

In Figure 5A, the performance metrics for Model 1 are presented in greater detail, encompassing accuracy, precision, specificity, and sensitivity percentage values. Notably, the P lung class exhibited the highest precision, accuracy, and sensitivity values which reached 90.5%, 89.8%, and 87.5%, respectively. The significance of the high degree of sensitivity lies in its direct correlation with the model’s ability to correctly detect true positive pathological tissues. Conversely, the class of C lungs attained the highest specificity values (Figure 5A). This highlights the superior ability of the model to distinguish false-positive C samples, thereby preventing the misclassification of N or P lungs as C, but not vice versa.

Following the external validation of Model 2 using the validation subset, the results were notably promising. Indeed, an overall precision of 90.5% in effectively identifying and categorizing the unlabeled pathological lung tissues as CBP, FPP, or IP was achieved (Table 4). Consequently, on a global scale, Model 2 demonstrated superior classification performance compared to Model 1. Remarkably, every lung in the IP category was correctly identified and none of the CBP or FPP samples were incorrectly classified as IP, resulting in 100% accuracy, precision, sensitivity, and specificity toward lung samples of the IP class (see Figure 5B). Model 2 also exhibited high accuracy, precision, and sensitivity rates for samples belonging to the CBP group (90.5%, 95.6%, and 92.2%, respectively), highlighting its remarkable ability/performance in distinguishing these samples from others (Table 4, Figure 5B). However, it is noteworthy that the precision value achieved for the FPP was found to be low (69.0%, Figure 5B). This outcome stems from the misclassification of nine FPP samples as CBP samples and the wrong recognition of five true CBP samples as FPP samples (Table 4).

## 4. Discussion

Although conducted as a preliminary step within a broader research project and characterized by its exploratory nature, this study yielded promising results concerning the application of NIR spectroscopy for discriminating categories of pig lung tissues. Indeed, it emerged that NIR spectral fingerprinting could successfully categorize N, C, and P pig lungs, as well as distinguish among specific lung inflammation patterns (CBP, FPP, and IP), opening new perspectives for more effective diagnostic and management strategies of porcine respiratory diseases by veterinarians.

Specifically, in this study, the first model to discriminate between N, C, and P pig lung samples was developed using the NIR spectra collected on these categories of tissues (Model 1). As shown in Figure 4A, distinct clusters for each tissue category were obtained. Notably, the three clusters tended to separate primarily along the first (horizontal) predictive component, indicating the potential ability of the model to perform such a task. However, some overlap was observed between samples from different categories. This observation underscores the complexity of tissue classification and suggests the presence of shared spectral characteristics among the studied tissues, resulting in overlapping features between categories in certain instances. It is crucial to acknowledge that the morphological diagnosis in this study was determined solely through macroscopic evaluation of the lungs by veterinarians. Therefore, it is possible that more detailed diagnostic information, such as those obtained by gold standard methods like histopathology, would have contributed to a more precise assignment of each sample to the correct tissue categories, thus improving model calibration and validation metrics. Indeed, given the complex pulmonary histoarchitecture and its inherent compositional inhomogeneity, the co-presence of variegate histological findings in adjacent lung lobules is quite common [34]. It is therefore plausible that within the lung area scanned with the portable NIR spectrometer, the replicate spectra could have been collected from lobules associated with different macroscopically indistinguishable lung tissue categories (i.e., in regions where N, C, and P tissue traits coexisted).

Generally, C lung tissue samples (Figure 4A) were positioned between N and P samples, suggesting intermediate spectral characteristics. Pulmonary congestion is characterized by increased blood volume in the lungs and can have several causes, including blood stasis due to cardiovascular disorders (passive hyperemia) and increased blood flow to injured tissue due to dilation of arterioles and capillaries during acute inflammation (active hyperemia) [35]. The increased amount of blood, and therefore of erythrocytes and macromolecules, may justify the separation of C from N samples, while their positioning between the N and P lung tissues may reflect the normal evolution of pneumonia.

Following the external validation of Model 1, the categorization of the P lung class exhibited the highest precision, accuracy, and sensitivity values. Indeed, tissue alterations characterizing acute lung inflammation, such as active hyperemia, edema, exudation, and leukocytic infiltration [36], may have resulted in a distinct and unique spectral fingerprint of P lungs, ultimately leading to the correct recognition of unlabeled P samples. In this context, it is crucial to note that attributing absorption peaks in the NIR region to particular chemical bonds and linking them to the molecules responsible for discrimination can be difficult. The VIP analysis for Model 1 revealed a highly intricate molecular fingerprint involved in distinguishing between N, C, and P tissues. As reported in Table 3, this fingerprint encompassed variations in amide, proteins, water, and hydrocarbon chains (typical of fatty acids) [33], which are related to tissue characteristics. These variations may potentially correlate with biochemical and cellular changes associated with tissue inflammation. However, further dedicated studies are needed to fully elucidate these relationships.

The second model was developed to discriminate between different patterns of pulmonary inflammation (Model 2). The three most common types of porcine pneumonia were considered, namely CBP, FPP, and IP. Compared to Model 1, the prediction ability of Model 2 was found to be higher. This outcome confirms the hypothesis that structural and compositional modifications in lung tissues, resulting from pathological processes, can be well captured within the NIR spectra. Moreover, it affirms the presence and usability of a substantial portion of NIR spectral variability associated with the specific pathological characteristics of lung tissues for proper sample recognition.

The score scatter plot of Model 2 showed distinct clusters for each pathological tissue category. A certain degree of overlap was evident between CBP and FPP samples (Figure 4B). While these two pneumonia patterns are typically taught as distinct entities, it is not uncommon in routine diagnostics to encounter mixed scenarios where typical findings of CBP coexist with FPP lesions. Consequently, for future endeavors, incorporating histopathological data remains imperative to achieve enhanced accuracy in model calibration. Notably, the separation of IP samples from CBP and FPP was particularly pronounced, and external validation resulted in 100% accuracy, precision, sensitivity, and specificity toward lung samples of the IP class (Figure 4B and Figure 5B). Indeed, pathological findings typical of lung interstitial inflammation are deeply different from those of other pneumonia models, and this may justify the results obtained. Whereas acute CBP and FPP are mainly characterized by exudation (catarrhal or fibrinous) and cellular infiltration (mainly neutrophils and macrophages) within the airspaces of the alveoli and distal airways, IP shows alterations primarily involving the peribronchiolar, perivascular, and perilobular interstitium. In acute cases, edema and abundant lymphoplasmacytic and histiocytic infiltrates expand alveolar and interlobular septa, while severe interstitial fibrosis is typical of chronic evolution [35]. When evaluating the NIR absorbance values which predominantly varied according to the type of pathological tissue and responsible for their discrimination (Table 3), a notable influence of water was observed, followed by amides/proteins [33]. This observation may suggest a potential relationship between exudation and cellular infiltration processes and the spectral fingerprints recorded in these types of lung lesions (Table 3). However, it is crucial for future studies to broaden the sample size, particularly within the IP category, to further substantiate these results. Additionally, complementing NIR analysis with other chemical techniques is essential for a comprehensive understanding of the samples under investigation.

Overall, the results of the present study provide a promising proof of concept regarding the use of NIR spectroscopy for the categorization of pig lung tissues and offer prospects for efficient diagnostic strategies. Indeed, the obtained performance metrics are notably satisfactory, aligning closely with those reported by other authors employing NIR spectroscopy for distinguishing between normal and pathological tissues. In fact, the accuracy of classification achieved in the present study reached levels greater than 90% and it is comparable to performance levels previously reported in the literature [26,27,28]. Additionally, based on the results, it appears that misclassifications were primarily linked to variations in the severity of pathology and a gradient from normal to pathological tissues. These observations are consistent with previous findings, highlighting the recurring challenge of tissue recognition across different studies [26,27,28].

In summary, the utilization of NIR spectroscopy, especially through the use of handheld spectrometers, holds significant potential not only in veterinary laboratories for routine diagnostics, but also in various other settings, particularly in abattoir inspection activities. For example, employing this technology for the systematic assessment of lung tissue characteristics in slaughtered pigs could yield extensive data for epidemiological studies and enhance on-farm animal health management practices. However, integrating a portable NIR instrument into a high-throughput industrial abattoir poses numerous challenges that require careful consideration [37]. In this regard, future research should explore the advancement of analytical sensors using contactless NIR spectroscopy principles. These sensors should be capable of analyzing tissue characteristics remotely and without the need for human operators, thus aligning with the requirements of Regulation (EU) 2019/627 on postmortem inspection in pig slaughterhouses [38], which calls for the application of a visual-only, risk-based meat quality assurance system [39].

Furthermore, the implementation of customized hyperspectral imaging systems operating in the NIR region within slaughter lines would represent a breakthrough innovation. These systems, based on the same principles as the portable NIR spectroscopic instrument employed in this study, operate within the same time frame as the portable instrument but can scan the entire organ surface and provide spatially resolved hyperspectral images. Additionally, NIR-based hyperspectral imaging systems, capturing information across a broader portion of the electromagnetic spectrum, offer advantages over conventional cameras, which operate within the visible light range (400–700 nm) and are increasingly being adopted in abattoir environments. Indeed, the broader spectrum would allow for better discrimination of artifacts, such as those arising from carcass geometry or the presence of blood, thus significantly enhancing the accuracy of lesion evaluation and control activities in abattoir settings.

## 5. Conclusions

The integration of advanced instrumental methods is rapidly emerging as a focal point in animal and veterinary sciences.

Despite the preliminary testing of NIR spectroscopy in a previously unexplored area of pig lung pathology assessment, the findings obtained in this study demonstrate the feasibility of using NIR spectroscopy for the automated and unbiased identification of normal, congested, and pathological pig lung tissues, as well as for the identification of specific pathological patterns. The non-destructiveness and rapidity of the method make it well suited for future integration along the slaughter line, allowing the semi-quantitative analysis of pig lung lesions and their severity. The classification models developed based on NIR spectra for lung tissue recognition hold significant potential as supporting tools for veterinarians conducting necropsies and gross diagnosis of respiratory diseases in pigs, facilitating efficient abattoir inspection activities. Such advancement has the potential to enhance disease management and prevention strategies, ultimately contributing to the improvement of both animal health and public health outcomes.

## Figures and Tables

**Table 1:** Distribution of NIR spectra across the various lung tissue classes.

Lung Tissue Class	No. of Collected NIR Spectra
N	419
C	291
CBP	451
FPP	113
IP	24

**Table 2:** Summary of the calibration performances of the discriminant models created.

Model	Components (Predictive + Orthogonal)	R^2^X_cum_	R^2^Y_cum_	Q^2^_cum_
Model 1 (N vs. C vs. P)	2 + 7	0.936	0.547	0.532
Model 2 (CBP vs. FPP. vs. IP)	2 + 7	0.904	0.647	0.615

**Table 3:** The ten most significant Variable Importance in Projection (VIP) values, highlighting the key NIR wavelengths for discriminating N vs. C vs. P (Model 1) and CBP vs. FPP vs. IP (Model 2) lung samples and their corresponding band assignments *.

Model 1 (N vs. C vs. P)			Model 2 (CBP vs. FPP vs. IP)		
NIR Wavelength (nm)	VIP Value	Assignment	NIR Wavelength (nm)	VIP Value	Assignment
1515	1.28	Amide/protein	1580	2.24	Alcohol/water
976	1.27	Water	1453	2.16	Water
970	1.24	-	1471	2.06	Amide/protein
964	1.23	Alkyl alcohol	1205	2.05	Water
1472	1.22	Aromatic amine	1463	2.03	Amide/protein
1521	1.22	Amide/protein	1212	1.95	Aliphatic hydrocarbons
1391	1.21	Aliphatic hydrocarbon	1023	1.95	Aromatic amines
1428	1.21	Primary amides	1218	1.62	Aliphatic hydrocarbons
1422	1.20	Aromatic hydrocarbon	1042	1.48	Aliphatic hydrocarbons
1397	1.20	Aliphatic hydrocarbon	1174	1.44	Alkenes

* Band assignment was performed according to [33].

**Table 4:** Confusion matrices depicting the allocation of lung samples within the considered lung classes for the external validation subsets of discriminant Models 1 and 2.

Model 1					Model 2				
Lung Tissue Class	Actual Members	N	C	P	Lung Tissue Class	Actual Members	CBP	FPP	IP
N	104	88	6	10	CBP	112	107	5	0
C	73	13	51	9	FPP	29	9	20	0
P	147	4	10	133	IP	6	0	0	6
Total	324	105	67	152	Total	147	116	23	6

## Data Availability

The raw data supporting the conclusions of this article will be made available by the authors upon request.
